# Using Administrative Data to Explore Potentially Aberrant Provision of Virtual Care During COVID-19: Retrospective Cohort Study of Ontario Provincial Data

**DOI:** 10.2196/29396

**Published:** 2021-09-07

**Authors:** Vess Stamenova, Cherry Chu, Andrea Pang, Mina Tadrous, R Sacha Bhatia, Peter Cram

**Affiliations:** 1 Institute for Health System Solutions and Virtual Care Women's College Hospital Toronto, ON Canada; 2 Institute for Clinical Evaluative Sciences Toronto, ON Canada; 3 Leslie Dan Faculty of Pharmacy University of Toronto Toronto, ON Canada; 4 Department of Medicine University of Toronto Toronto, ON Canada; 5 School of Medicine University of Texas Medical Branch Galveston, TX United States

**Keywords:** telemedicine, virtual care, COVID-19, pandemic, virtual health, telehealth, ambulatory visits, physicians, patients, digital health

## Abstract

**Background:**

The COVID-19 pandemic has led to a rapid increase in virtual care use across the globe. Many health care systems have responded by creating virtual care billing codes that allow physicians to see their patients over telephone or video. This rapid liberalization of billing requirements, both in Canada and other countries, has led to concerns about potential abuse, but empirical data are limited.

**Objective:**

The objectives of this study were to examine whether there were substantial changes in physicians’ ambulatory visit volumes coinciding with the liberalization of virtual care billing rules and to describe the characteristics of physicians who significantly increased their ambulatory visit volumes during this period. We also sought to describe the relationship between visit volume changes in 2020 and the volumes of virtual care use among individual physicians and across specialties.

**Methods:**

We conducted a population-based, retrospective cohort study using health administrative data from the Ontario Health Insurance Plan, which was linked to the ICES Physician Database. We identified a unique cohort of providers based on physicians’ billings and calculated the ratio of total in-person and virtual ambulatory visits over the period from January to June 2020 (virtual predominating) relative to that over the period from January to June 2019 (in-person predominating) for each physician. Based on these ratios, we then stratified physicians into four groups: low-, same-, high-, and very high–use physicians. We then calculated various demographic and practice characteristics of physicians in each group.

**Results:**

Among 28,383 eligible physicians in 2020, the mean ratio of ambulatory visits in January to June 2020:2019 was 0.99 (SD 2.53; median 0.81, IQR 0.59-1.0). Out of 28,383 physicians, only 2672 (9.4%) fell into the high-use group and only 291 (1.0%) fell into the very high–use group. High-use physicians were younger, more recent graduates, more likely female, and less likely to be international graduates. They also had, on average, lower-volume practices. There was a significant positive correlation between percent virtual care and the 2020:2019 ratio only in the group of physicians who maintained their practice (*R*=0.35, *P*<.001). There was also a significant positive correlation between the 2020:2019 ratio and the percent virtual care per specialty (*R*=0.59, *P*<.01).

**Conclusions:**

During the early stages of the pandemic, the introduction of virtual care did not lead to significant increases in visit volume. Our results provide reassuring evidence that relaxation of billing requirements early in the COVID-19 pandemic in Ontario were not associated with widespread and aberrant billing behaviors. Furthermore, the strong relationship between the ability to maintain practice volumes and the use of virtual care suggests that the introduction of virtual care allowed for continued access to care for patients.

## Introduction

The COVID-19 pandemic has led to a rapid increase in virtual care use across the globe [1-5]. In Ontario, Canada’s largest province, virtual care increased from 1.6% of all ambulatory visits pre–COVID-19 to 71% during the first wave of the COVID-19 pandemic [3], a much higher rate compared to those reported in other countries such as the United States (30%) and Australia (42%) [4,6,7].

While Ontario had pre-existing virtual care billing codes before the onset of the pandemic, these codes were allowable for a single government-run online platform and only available to specialists and primary care physicians in rostered patient practices or specialized practices. Primary care physicians outside of rostered practices were not included in this model, in order to support continuity of care [8] and respond to growing concerns about fragmentation and poor quality of care received in virtual walk-in clinics as well as funding disruptions in Canada [9] and abroad [10].

In Ontario, the pandemic led to the introduction of temporary billing codes in mid-March 2020 that reimbursed any physician with identical amounts for in-person, video, or telephone visits and eliminated prior restrictions on practice type or allowable technology platforms. This rapid liberalization of billing requirements, both in Canada and other countries [4], has led to concerns about potential abuse, but empirical data are limited [11].

The objectives of this study were to examine whether there were substantial changes in physicians’ ambulatory visit volumes coinciding with the liberalization of virtual care billing rules and to describe the characteristics of physicians who significantly increased their ambulatory visit volumes during this period. We also sought to describe the relationship between visit volume changes in 2020 and the volumes of virtual care use in individual physicians and across specialties.

## Methods

We conducted a population-based, retrospective cohort study using health administrative data from the Ontario Health Insurance Plan, which was linked to the ICES Physician Database. Data sets were linked using unique encoded identifiers and analyzed at ICES, an independent, nonprofit research institute. Use of these databases for the purposes of this study was authorized under §45 of Ontario’s Personal Health Information Protection Act, which does not require review by a research ethics board. An exemption letter was obtained by the Research Ethics Board at Women’s College Hospital, Toronto, Ontario.

We identified a unique cohort of providers based on physicians’ billings for in-person and virtual ambulatory visits. We excluded visits for non-Ontario residents and those with an invalid or missing health card number. We also excluded all physicians with clinical volumes that were inconsistent with an active practice during the pre–COVID-19 period (<10 ambulatory visits during the period from January to June 2019).

We then calculated the ratio of total in-person and virtual ambulatory visits over the period from January to June 2020 (virtual predominating) relative to that over the period from January to June 2019 (in-person predominating) for each physician. We included first-quarter data in 2020 as they cover the beginning of the pandemic. Data extending past the second quarter of 2020 were unavailable. Based on these ratios, we then stratified physicians into four groups: (1) low-use physicians had ratios from 0 to 0.50 (ie, a 50% or greater reduction in visits in 2020 compared to 2019), (2) same-use physicians were those with ratios over 0.50 but less than 1.25, (3) high-use physicians were those with ratios of at least 1.25 but less than 6.0, and (4) very high–use physicians were those with ratios equal to or greater than 6.0 (ie, an at-least 6-fold increase in visits in 2020 compared to 2019). To explore whether the proportions of physicians falling into each category differed much from previous years, we also calculated the number of physicians falling into each group—defined as the same ratio ranges—for the periods of January to June 2019 relative to January to June 2018.

For all physicians, we also obtained demographic and practice characteristics, including, age, sex, years since graduation, training location, practice type (ie, specialist, family practice, or focused family practice physicians focusing 50% or more of their practice in a specific type of care, such as psychotherapy [12]), and specialty. We also calculated the number of unique patients seen, number of total visits, number of virtual visits, and number of visits per day, virtual or any.

## Results

**Figure 1:**
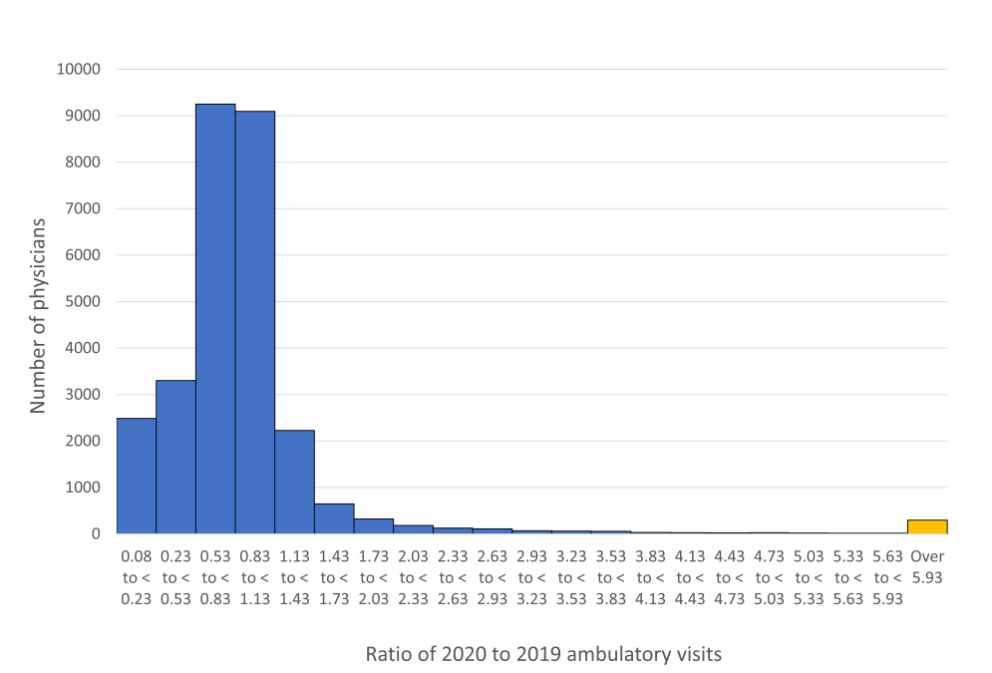
Histogram showing the total number of physicians by ambulatory visit volume ratio (2020:2019). Proportions of <1 indicate fewer visits in the period of January to June 2020 when compared to January to June 2019, while a proportion of >1 suggested increased visits in the period of January to June 2020. Note, the last bin in orange represents anybody with a ratio over 5.93, representing the top 1%.

Among 28,383 eligible physicians in 2020, the mean ratio of ambulatory visits in January to June 2020:2019 was 0.99 (SD 2.53; median 0.81, IQR 0.59-1.0). Only 291 physicians (1.0%) were very high users, 2672 physicians (9.4%) were high users, and 5422 (19.1%) were low users (Figure 1). In comparison, the previous year (2019:2018 visit ratio), among 27,709 eligible physicians, 289 (1.0%) were very high users, 3395 (12.3%) were high users, and 2937 (10.6%) were low users.

High-use physicians were younger, more recent graduates, more likely to be female, and less likely to be international graduates than those who maintained their volumes (ie, same-use group) (*P*<.001) (Table 1). These effects were even more pronounced in the very high–use group. They were also more likely to be specialists than primary care providers (*P*<.001), and physicians in focused primary care practices were more likely to be high or very high users relative to specialists (*P*<.001).

Physicians in the two high-use groups had, on average, lower-volume practices in both 2020 and 2019 (*P*<.001), and volumes were especially smaller in 2019 with fewer patients seen (*P*<.001). The effect was larger for the very high users (*P*<.001). Providers in the two high-use groups had fewer visits per billing day and fewer total patients seen in 2020 (*P*<.001), but they had a higher percentage of virtual care visits (*P*<.001) and a similar number of virtual care visits per day compared to the same-use group (Table 1).

Specialties with a large percentage of their total physician population being in the high-use group included emergency medicine (41/230, 17.8%), psychiatry (378/2061, 18.3%), and internal medicine (147/999, 14.7%). The same specialties were common among the very high–use group (Table 2).

**Figure 2:**
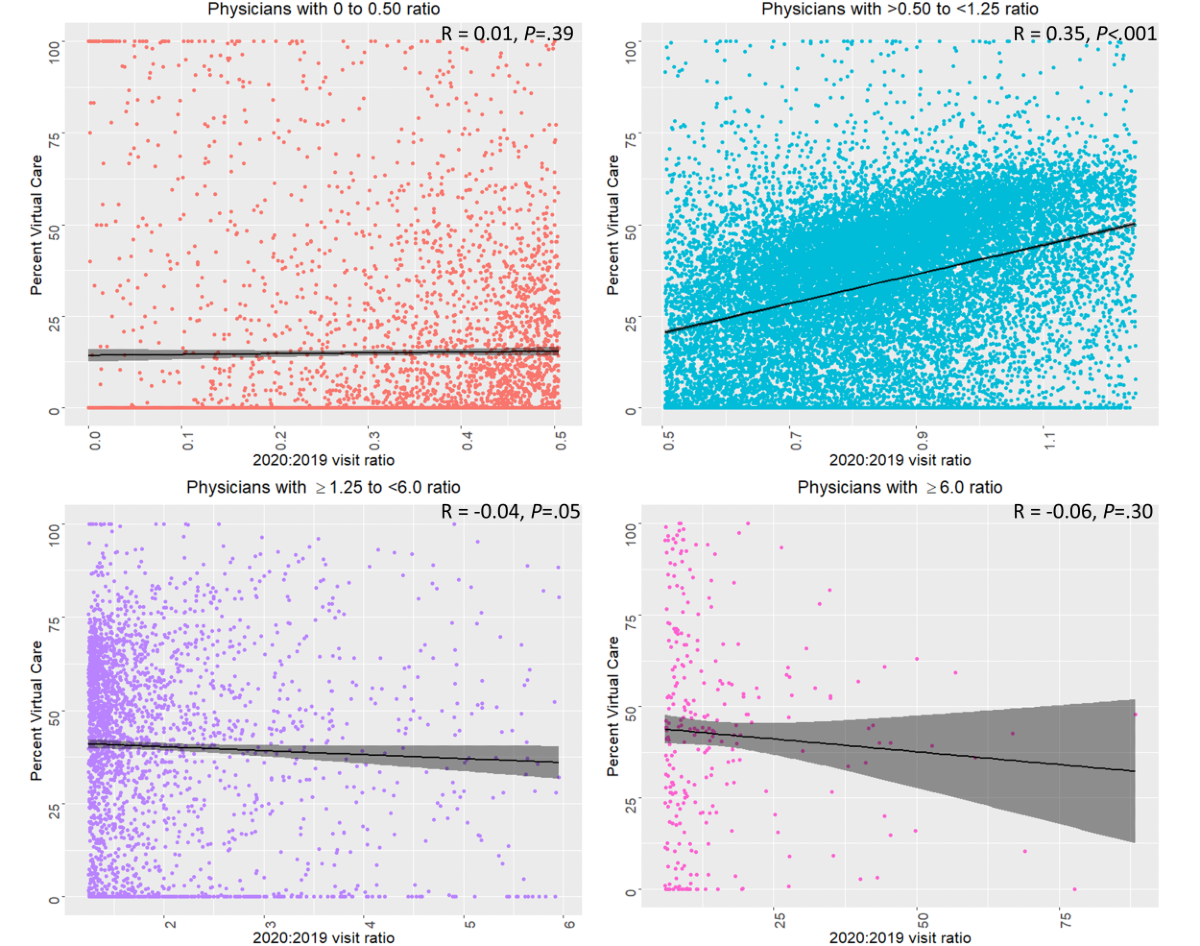
Correlation between the 2020:2019 visit ratio and percent virtual care in the four groups of providers: those who reduced (0 to 0.50), maintained (>0.5 to <1.25), increased (1.25 to <6), and significantly increased (≥6) their practice in 2020 relative to 2019.

Pearson correlations between the 2020:2019 visit ratio and percent of visits completed virtually showed a significant positive correlation only among physicians from the same-use group (*R*=0.35, *P*<.001) (Figure 2).

**Figure 3:**
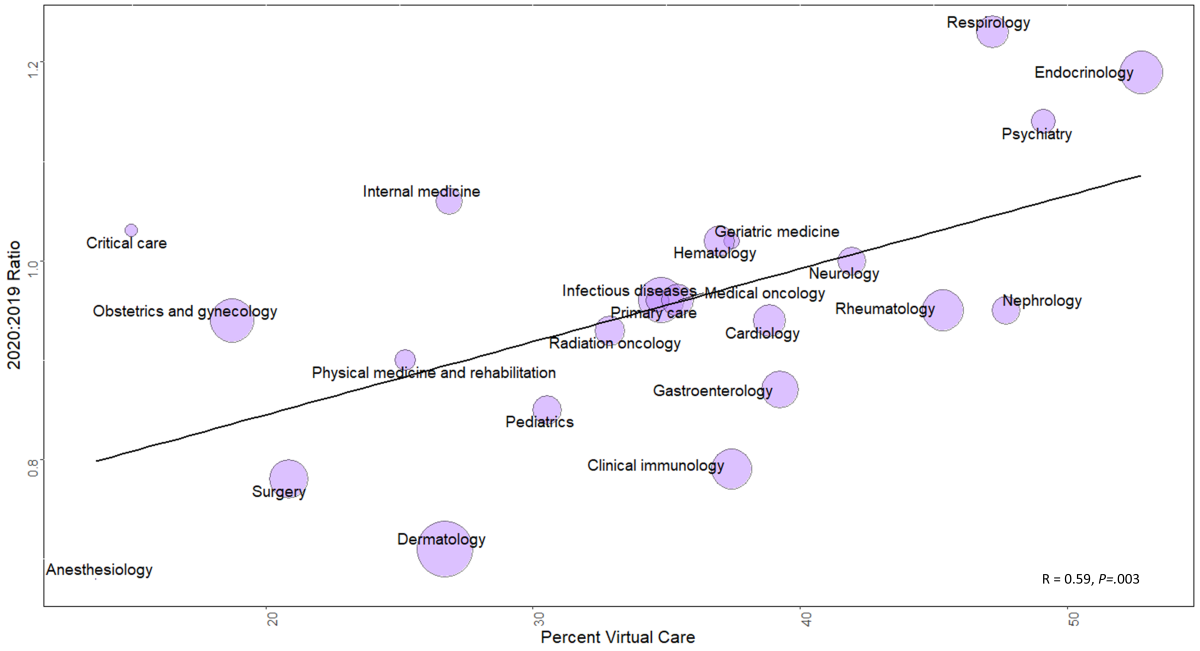
Correlation between the 2020:2019 visit ratio and virtual care adoption across specialties. The size of each sphere indicates the number of visits completed.

Finally, we also calculated the Pearson correlation coefficient between the average 2020:2019 ratio per specialty and the percent virtual care used per specialty. We excluded emergency medicine, diagnostic radiology, and nuclear medicine, as they were outliers and had both the highest 2020:2019 visit ratios (1.6, 1.3, and 2.9, respectively) and the lowest percentages of virtual care (12.4%, 1.5%, and 8.2%, respectively). There was a significant positive correlation between the 2020:2019 ratio and the percent virtual care across specialties (*R*=0.59, *P*<.01) (Figure 3).

**Table 1:** Physician characteristics stratified by ratio of ambulatory visits in January to June 2020:2019.

Characteristic		Physicians					*P* value
		All (N=28,383)	Low use: 0 to 0.50 visits^a^ (n=5422)	Same use: >0.50 to <1.25 visits (n=19,998)	High use: 1.25 to <6.0 visits^b^ (n=2672)	Very high use: ≥6.0 visits (n=291)	
Age (years), mean (SD)		53.9 (11.2)	56.1 (12.6)	53.7 (10.8)	51.9 (11.24)	49.2 (11.5)	<.001
Years since graduation, mean (SD)		25.1 (13.3)	26.7 (15.5)	25.4 (12.5)	20.5 (12.60)	15.8 (11.7)	<.001
Sex (female), n (%)		11,864 (41.8)	2287 (42.5)	8116 (40.6)	1268 (48.4)	148 (55.4)	<.001
Canadian or internationally trained (Canadian trained), n (%)		16,218 (76.4)	2928 (77.2)	11,912 (75.7)	1282 (81.6)	96 (83.5)	<.001
**Number of unique patients seen in 2019 (January to June)**							
	Mean (SD)	729.2 (795.0)	496.53 (675.0)	858.28 (828.8)	308.81 (433.4)	55.46 (60.5)	<.001
	Median (IQR)	539 (163-999)	257 (75-689)	691 (295-1129)	153 (50-402)	35 (17-72)	<.001
**Number of unique patients seen in 2020 (January to June)**							
	Mean (SD)	556.7 (618.1)	155.2 (297.0)	684.5 (639.5)	428.0 (577.4)	439.3 (408.9)	<.001
	Median (IQR)	408 (100-789)	35 (0-183)	559 (241-910)	236 (81-567)	347 (133-590)	<.001
**Percent virtual care visits in 2020 (January to June)**							
	Mean (SD)	31.9 (22.7)	14.95 (24.3)	34.03 (20.0)	40.3 (26.2)	42.3 (28.6)	<.001
	Median (IQR)	34 (11-49)	0 (0-22)	37 (19-49)	44 (18-60)	42 (21-61)	<.001
**Number of virtual care visits in 2020 (January to June)**							
	Mean (SD)	400.7 (571.0)	52.1 (139.5)	471.3 (570.6)	411.9 (771.3)	294.8 (315.1)	<.001
	Median (IQR)	231 (30-551)	1 (0-37)	324 (98-639)	199 (32-494)	205 (70-432)	<.001
**Number of total visits in 2020 (January to June)**							
	Mean (SD)	1101.2 (1228.9)	292.2 (484.9)	1300.0 (1257.7)	877.9 (1293.1)	738.0 (655.7)	<.001
	Median (IQR)	780 (240-1503)	108 (23-370)	1004 (454-1715)	495 (151-1105)	538 (274-976)	<.001
**Number of total visits in 2019 (January to June)**							
	Mean (SD)	1304.8 (1420.4)	800.5 (1103.6)	1561.5 (1483.3)	541.4 (855.9)	66.1 (73.7)	<.001
	Median (IQR)	934 (286-1799)	392 (101-1128)	1212 (539-2078)	267 (74-658)	43 (20-84)	<.001
**Number of visits per billing day in 2020 (January to June)**							
	Mean (SD)	12.1 (9.5)	8.1 (8.3)	13.2 (9.5)	9.7 (9.8)	10.2 (7.3)	<.001
	Median (IQR)	9 (5-15)	5 (2-10)	11 (6-16)	7 (3-12)	7 (4-12)	<.001
**Number of virtual visits per billing day in 2020 (January to June)**							
	Mean (SD)	8.6 (6.7)	5.0 (5.2)	9.1 (6.5)	8.1 (7.6)	7.4 (5.4)	<.001
	Median (IQR)	7 (4-11)	3 (2-6)	7 (4-11)	6 (3-10)	6 (3-9)	<.001
**Practice type, n (%)**							
	Specialist	15,201 (53.6)	3058 (56.4)	10,419 (52.1)	1590 (59.5)	134 (46.0)	<.001
	Primary care provider	9393 (33.1)	1366 (25.2)	7560 (37.8)	436 (16.3)	31 (10.7)	N/A^c^
	Focused primary care provider^d^	426 (1.5)	120 (2.2)	190 (1.0)	97 (3.6)	19 (6.5)	N/A
	Miscellaneous	3363 (50.0)	878 (13.1)	1829 (27.2)	549 (8.2)	107 (1.6)	N/A

^a^Visits of 0 to 0.50 correspond to physicians who had a 50% or greater reduction in ambulatory visits between 2019 and 2020.

^b^Visits of 1.25 to <6 correspond to physicians who had a 25% 6-fold increase in visits between 2019 and 2020.

^c^N/A: not applicable; a single test was conducted across all four groups in this section and the *P* value is reported in the row for the first group.

^d^Focused primary care providers are primary care providers who specialize in a specific care (eg, palliative care).

**Table 2:** Physicians per specialty across each user group.

Specialty		Low use: 0 to 0.5, n (%)	Same use: >0.5 to <1.25, n (%)	High use: 1.25 to <6.0, n (%)	Very high use: ≥6.0, n (%)
Family medicine (n=13,244)		2366 (17.9)	9715 (73.4)	1042 (7.9)	121 (0.9)
**Medicine**					
	Emergency medicine (n=230)	57 (24.8)	126 (54.8)	41 (17.8)	6 (2.6)
	Internal medicine (n=999)	233 (23.3)	604 (60.5)	147 (14.7)	15 (1.5)
	Infectious diseases (n=148)	27 (18.2)	101 (68.2)	20 (13.5)	0 (0)
	Critical care (n=99)	29 (29.3)	53 (53.5)	13 (13.1)	≤5 (≤5.1)
	Endocrinology (n=269)	18 (6.7)	215 (79.9)	34 (12.6)	≤5 (≤1.9)
	Nuclear medicine (n=48)	15 (31.3)	24 (50.0)	6 (12.5)	≤5 (≤10.4)
	Hematology (n=217)	23 (10.6)	164 (75.6)	27 (12.4)	≤5 (≤2.3)
	Cardiology (n=684)	77 (11.3)	527 (77.0)	78 (11.4)	≤5 (≤0.7)
	Respirology (n=308)	32 (10.4)	237 (76.9)	35 (11.4)	≤5 (≤1.6)
	Geriatric medicine (n=152)	29 (19.1)	105 (69.1)	16 (10.5)	≤5 (≤3.3)
	Rheumatology (n=213)	19 (8.9)	172 (80.8)	22 (10.3)	0 (0)
	Anesthesiology (n=1179)	480 (40.7)	597 (50.6)	101 (8.6)	≤5 (≤0.4)
	Nephrology (n=242)	23 (9.5)	198 (81.8)	20 (8.3)	≤5 (≤2.1)
	Clinical immunology (n=84)	22 (26.2)	56 (66.7)	6 (7.1)	0 (0)
	Gastroenterology (n=345)	39 (11.3)	281 (81.4)	24 (7.0)	≤5 (≤1.4)
	Obstetrics and gynecology (n=808)	98 (12.1)	659 (81.6)	45 (5.6)	6 (0.7)
**Other specialties**					
	Psychiatry (n=2061)	328 (15.9)	1326 (64.3)	378 (18.3)	29 (1.4)
	Diagnostic radiology (n=624)	179 (28.7)	355 (56.9)	84 (13.5)	6 (1.0)
	Medical oncology (n=269)	34 (12.6)	200 (74.3)	35 (13.0)	0 (0)
	Pediatrics (n=1487)	395 (26.6)	938 (63.1)	146 (9.8)	8 (0.5)
	Radiation oncology (n=211)	14 (6.6)	178 (84.4)	19 (9.0)	0 (0)
	Neurology (n=410)	53 (12.9)	321 (78.3)	33 (8.0)	≤5 (≤1.2)
	Physical medicine and rehabilitation (n=211)	48 (22.7)	146 (69.2)	16 (7.6)	≤5 (≤2.4)
	Dermatology (n=236)	52 (22.0)	173 (73.3)	11 (4.7)	0 (0)
	Surgery (n=2868)	536 (18.7)	2212 (77.1)	104 (3.6)	16 (0.6)
	Remaining smaller specialties (n=384)	91 (23.7)	246 (64.1)	43 (11.2)	4 (1.0)
	Miscellaneous (n=353)	105 (29.7)	69 (19.5)	126 (35.7)	53 (15.0)

## Discussion

During the early stages of the pandemic, the introduction of virtual care did not lead to significant increases in visit volume. Only about 10% of physicians increased their visit volumes by 25% or more in 2020 relative to 2019. In total, our results provide reassuring evidence that relaxation of billing requirements early in the COVID-19 pandemic in Ontario were not associated with widespread and aberrant billing behaviors.

Providers who increased their visit volumes tended to be specialists, younger, more recent graduates, and more likely female. Among providers who increased their practice volumes, there was no relationship between the magnitude of increase and virtual care adoption. A significant relationship was observed, however, among providers who maintained their practice. This relationship was also maintained at the specialty level. Endocrinology, respirology, and psychiatry maintained their practices the best and had higher rates of virtual care adoption.

Our results are consistent with data from the United States that showed that despite the introduction of virtual care, overall visit volumes decreased in the early periods of the COVID-19 pandemic [6]. In fact, the introduction of virtual care during the pandemic allowed physicians to maintain their practices. Higher rates of virtual care use among providers who maintained their practice volumes were associated with better maintenance of visit volumes during the pandemic. This trend was also observed in the United States [6]. Here, we confirm these findings with an analysis of the entire physician and patient population in a health care system with a single insurance plan where the introduction of virtual care payment policies occurred at the same time for the entire population.

At least two specialties that showed high virtual care adoption rates and good maintenance of visit volumes during the pandemic were consistent in both Ontario and the United States [6]: psychiatry and endocrinology. Mental health care has the potential to be better suited for virtual care as it often does not require a physical exam and it has been successful in adopting virtual care services both before [13] and after the pandemic [14]. Successful adoption in endocrinology during the pandemic has also been reported [15].

Limitations to our study include a relatively brief time window for evaluating the impact of billing code liberalization, which makes it unclear whether the trends will be maintained in the long term. Our reliance on administrative data also precludes us from robustly evaluating appropriateness of individual visits.

In total, our study suggests that liberalization of virtual care billing requirements coinciding with the COVID-19 pandemic was not associated with an alarming increase in individual physician visit volumes and should serve to assuage concerns over widespread fraud. Furthermore, the strong relationship between the ability to maintain practice volumes and the use of virtual care suggest that the introduction of virtual care allowed continued access to care for patients.
